# Assessing Driving Risk at the Second Phase of Overtaking on Two-Lane Highways for Young Novice Drivers Based on Driving Simulation

**DOI:** 10.3390/ijerph19052691

**Published:** 2022-02-25

**Authors:** Jie Pan, Yongjun Shen

**Affiliations:** 1 School of Transportation, Southeast University, Nanjing 210096, China; panj21@mails.tsinghua.edu.cn; 2 Institute of Transportation Engineering, Tsinghua University, Beijing 100089, China

**Keywords:** overtaking on two-lane highways, back-to-lane phase, young novice drivers, risk assessment, driving simulation

## Abstract

Overtaking on two-lane highways is a complex and multi-phase maneuver associated with high collision risk, especially for young novice drivers. Most of the relevant studies, however, focused mainly on the first phase, i.e., the lane-changing phase, such as willingness to overtake, while the second phase, i.e., the back-to-lane phase, has not been investigated systematically. It is a risky phase in which a driver faces the risk of collision with not only the approaching vehicle on the opposite lane but also the impeding vehicle at the original lane. In this study, by designing and conducting a driving simulator experiment, we assess the driving risk of 47 young novice drivers during their second phase of overtaking on two-lane highways. The time-to-collision (TTC) values at the two critical positions are calculated from a micro-geometric point of view, based on which a two-dimensional risk index is proposed and the fuzzy C-means clustering algorithm is applied to group all the samples and to assess their overtaking risk. Furthermore, a multi-class logistic model is developed to understand the potential factors related to the risky overtaking maneuvers at this phase. The results show that most of the young novice drivers cannot make accurate judgments during their second phase of overtaking. When turning back to the original lane, they are more likely to be aware of the opposite vehicle that is approaching them, while how to correctly avoid the collision risk with the impeding vehicle at this phase is probably a more critical issue for young novice drivers.

## 1. Introduction

Overtaking is a common traffic phenomenon. Its intention mainly comes from the speed difference between the main vehicle and other vehicles, as well as the tendency of each driver to maintain the desired speed during driving [1]. From a psychological point of view, a decision on overtaking or not involves choosing between an undesirable choice (stay) and a risky choice (overtaking). Both choices have a satisfactory result (success) and a highly unwanted result (failure or a crash) [2]. Extensive attention has been paid to this driving scenario in the literature [3,4,5,6,7,8]. Now, when the overtaking maneuver is to be performed on a two-lane highway, the crash risk is increased, because a driver needs to drive on the opposite lane, which results in a possibility to collide with not only the impeding vehicle but also the opposite vehicle. In the whole process, the driver may a priori or concurrently have to make complex decisions requiring increased mental workload [9], which is particularly difficult for young novice drivers. Research by Mohayany et al. [10] showed that younger drivers (18–28 years old) are more likely to make mistakes when overtaking. In annual overtaking-related road crashes, young novice drivers account for a relatively large proportion, which is twice as high as that of elder and more experienced counterparts [11,12,13].

Wilson and Best [14] have divided the task of overtaking in two-lane highways into five phases: (i) deciding whether to overtake or not, (ii) preparing to overtake, (iii) changing lane, (iv) passing, and (v) returning to own lane. Later work simplified five-stage models for overtaking, with a three-stage model consisting of ‘lane-change’, ‘overtake’, and ‘merge’, becoming the most commonly used model to study overtaking maneuvers [15,16]. Vlahogianni [17] even further reduced overtaking maneuvers, resulting in only two stages.

**Figure 1:**
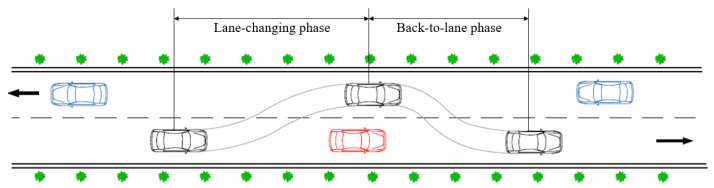
Two phases of overtaking.

In general, the overtaking maneuver on a two-lane highway can be divided into two phases (see Figure 1):

- The lane-changing phase, in which the driver decides to overtake and then changes to the opposite lane.
- The back-to-lane phase, in which the driver returns from the opposite lane to the original lane.

Most of the previous studies on two-lane overtaking focus on the first phase. Moreover, the risk in this phase has been systematically investigated from two aspects: (1) observation of critical safety indicators (e.g., following gap distance, passing sight distance, speed, and time); (2) factors affecting the critical time point of overtaking. Different methods and data collection techniques were applied for these purposes, such as video analysis [1,18], the use of instrumented vehicles equipped with different types of sensors [19,20], or driving simulation [21,22,23,24]. Figueira et al. [24] used the following gap distance as an indicator of the crash risk towards the impeding vehicle. Some scholars have studied the risk towards the opposite vehicle, using for instance the passing sight distance model [25,26] and the time-to-collision model [27,28]. The influencing factors of overtaking risk are very complex, involving people, vehicles, and road environments. Mocsari [29] compared the differences between two overtaking strategies at the beginning of the maneuver, the length of one overtaking, the overtaking time, the relative speed, the overtaking speed at the end of the maneuver, and the distance back to the lane. It was found that the type and speed of the impeding vehicle were the factors that most strongly influence the time and distance traveled on the opposite lane. Farah [30] revealed that differences in age and gender have a large influence on the frequency of overtaking maneuvers. Shinar [31] studied the differences of overtaking in driving styles, such as aggressive driving. Rahul and Vinod [18] considered vehicle type, road type, the relative speed of overtaking and being overtaken, and other factors when studying overtaking habits of Indian drivers. Results indicated that the presence of oncoming vehicles and the type of overtaken vehicle had a large and statistically significant impact on overtaking. 

Although the literature is extensive on the risk evaluation of overtaking on two-lane highways and the factors that may affect the critical gaps for overtaking, as well as the willingness to overtake, most of them are related to the first phase of overtaking. The second phase, however, has not been investigated systematically. At this phase, the driver of the vehicle faces not only the risk of collision with the approaching vehicle on the opposite lane but also the risk of collision with the impeding vehicle at the original lane. Therefore, multi-risk assessment is required. In this study, by collecting driving behavior data of 47 young novice drivers from a driving simulation experiment, the time-to-collision (TTC) values at the two critical positions are calculated from a micro-geometric point of view, and they are used as multi-risk indications of the driver who is performing the second phase of the overtaking maneuver. Then, the fuzzy C-means clustering algorithm is applied to group all these drivers under study and to assess their overtaking risk. Thereafter, a multi-class logistic model is built to reveal the critical factors associated with the risky overtaking maneuvers at this phase.

The rest of this paper is organized as follows. Section 2 introduces the methodology we proposed in this study to assess the driving risk at the second phase of overtaking on two-lane highways. Section 3 elaborates the simulator experiment we designed and conducted for this study, and the data collected from this experiment. The results on risk assessment and factor analysis are presented in Section 4, followed by a discussion section regarding the experiment design and data analysis. The paper ends with some interesting findings from this study as well as directions for future research.

## 2. Risk Assessment for the Second Phase of Overtaking

### 2.1. Overtaking Collision Types

In the past, scholars usually evaluated the risk of overtaking according to a certain risk point in the whole process, but rarely studied each type of collision and its influencing factors.

**Figure 2:**
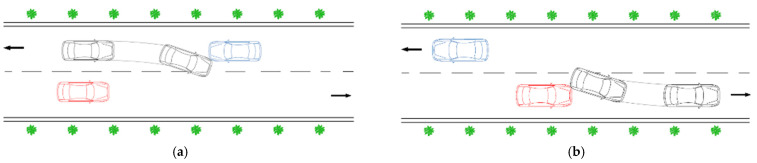
Collision types during the second phase of overtaking on a two-lane highway. (**a**) A collision with the opposite vehicle; (**b**) a collision with the impeding vehicle when turning back to the original lane.

Taking various situations during overtaking into account, the risk at the second phase of overtaking on a two-lane highway can be generally divided into the following two categories, as shown in Figure 2.

Figure 2a shows a critical position where the subject vehicle returns to its original lane too late, thus it collides with the opposite vehicle, and Figure 2b shows another critical position at which a collision occurs between the subject vehicle and the impeding vehicle when the subject vehicle turns back too early. During the second phase of overtaking, either of these two situations should be avoided. Thus, we propose in this study a two-dimensional risk index by computing the TTC value at each of these two critical points, so as to measure the driving risk of a set of novice drivers during this phase. 

### 2.2. TTC at the Two Critical Positions

**Figure 3:**
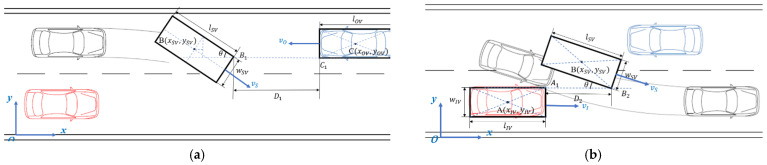
Variables of critical position (**a**,**b**) for calculating TTC.

The specific definition of TTC is the time to a collision when two vehicles continue to drive at the current speed without modifying trajectory, which is an important surrogate safety indicator to measure the risk of a conflict [32]. During the second phase of overtaking, when the opposite vehicle is approaching, the subject vehicle will turn the steering wheel back to its original lane. When its front left corner $\left(B_{1}\right)$ is at the same *y* coordinate as the left side $\left(C_{1}\right)$ of the opposite vehicle, it comes to the critical position (a), as shown in Figure 3.

Assume that the geometric center point of the opposite vehicle is:

(1) $$C(x_{OV},y_{OV})$$

The length of the opposite vehicle $\left(l_{OV}\right)$, the width of the opposite vehicle $\left(w_{OV}\right)$, the length of the subject vehicle $\left(l_{SV}\right)$ and the width of the subject vehicle $\left(w_{SV}\right)$ are already known. Thus, the coordinate of the front left corner of the opposite vehicle, i.e., $C_{1}$, can be expressed as follows:

(2) $$C_{1}(x_{OV}-\frac{l_{OV}}{2},y_{OV}-\frac{w_{OV}}{2})$$

where $l_{OV}$ and $w_{OV}$ represent the length and width of the opposite vehicle, respectively.

Moreover, the coordinate of the front left corner of the subject vehicle, i.e., $B_{1}$ is as follows:

(3) $$B_{1}\left(x_{SV}+\frac{l_{SV}cos\theta +w_{SV}sin\theta }{2},y_{SV}+\frac{w_{SV}cos\theta -l_{SV}sin\theta }{2}\right)$$

where $l_{SV}$ and $w_{SV}$ represent the length and width of the subject vehicle, respectively, and θ is the rotating angle of the subject vehicle. 

Let the y coordinates of $B_{1}$ and $C_{1}$ be equal:

(4) $$y_{OV}-\frac{w_{OV}}{2}=y_{SV}+\frac{w_{SV}cos\theta -l_{SV}sin\theta }{2}$$

Thus, the critical distance at this moment can be obtained:

(5) $$D_{1}=x_{OV}-\frac{l_{OV}}{2}-x_{SV}-\frac{l_{SV}cos\theta +w_{SV}sin\theta }{2}$$

The $TTC_{a}$ can then be calculated as follows:

(6) $$TTC_{a}=\frac{D_{1}}{v_{S}cos\theta +v_{O}}$$

where $v_{S}$ and $v_{O}$ are the instantaneous speeds of the two vehicles at the moment. 

Similar to the critical position (a), when the front right corner $\left(B_{2}\right)$ of the subject vehicle is at the same y coordinate as the left side $\left(A_{1}\right)$ of the impeding vehicle, it comes to the critical position (b).

The critical distance at this moment can be obtained:

(7) $$D_{2}=x_{SV}-\frac{l_{IV}}{2}-x_{IV}+\frac{l_{SV}cos\theta -w_{SV}sin\theta }{2}$$

Thus, the corresponding $TTC_{b}$ is given as follows:

(8) $$TTC_{b}=\frac{D_{2}}{v_{S}cos\theta -v_{I}}$$

Based on the value of $TTC_{a}$ and $TTC_{b}$ computed from Equations (6) and (8), a two-dimensional risk index can be developed, which can be used to measure the driving risk of a driver during his/her second phase of overtaking on a two-lane highway.

### 2.3. Overtaking Risk Classification Based on Fuzzy C-Means Clustering (FCM)

FCM is a clustering algorithm based on partition. Its idea is to maximize the similarity between objects that are divided into the same cluster and to minimize the similarity between different clusters. The fuzzy C-means clustering is an improvement of the ordinary C-means clustering by avoiding strict division of each object into a certain category, that is, FCM allows to establish a sample’s uncertain description of a category, which can reflect the process of overtaking more objectively.

The objective function of the FCM algorithm is:

(9) $$J_{m}\left(U,V\right)=\overset{c}{\underset{i=1}{\sum }}\overset{n}{\underset{j=1}{\sum }}u_{ij}^{m}{||X_{j}-V_{i}||}_{A}^{2}$$

where *U* = [$u_{ij}$] is the membership degree matrix, $u_{ij}$ is the membership degree of the $j^{th}$ sample for the $i^{th}$ category, and *m* is the fuzzy constant.

There are two critical collision points at the second phase of overtaking, namely critical positions (a) and (b). In this study, to comprehensively evaluate the crash risk at this phase, we calculate the risk index TTC of these two critical points and apply the FCM algorithm to group the drivers based on their two-dimensional risk index.

### 2.4. Analysis of Influencing Factors

Having classified the drivers based on their two-dimensional risk index, the potential influencing factors can be further investigated, which is conducted by applying a multi-class logistic regression model in this study.

The multi-class logistic regression model is an extension of the two-class logistic regression model. For all *K* possible classification results, in the process of running *K*−1 independent two-class logistic regression models, one of the categories is regarded as the main category. The remaining *K*−1 categories and the main categories are regressed separately. We select *K* as the main category here, and the logistic function shown in Equation (10) can be obtained:

(10) $$ln\frac{P\left(y=K-1|X\right)}{P\left(y=K|X\right)}=k_{n,K-1}x_{n}$$

The probability that the dependent variable will take a certain result under the condition of a given unpredicted sample is calculated as follows:

(11) $$p_{K-1}=P\left(y=K-1|X\right)=\frac{e^{k_{n,K-1}x_{n}}}{1+\sum_{m=1}^{K-1}e^{k_{n,m}x_{n}}}$$

## 3. The Experiment and Data

**Figure 4:**
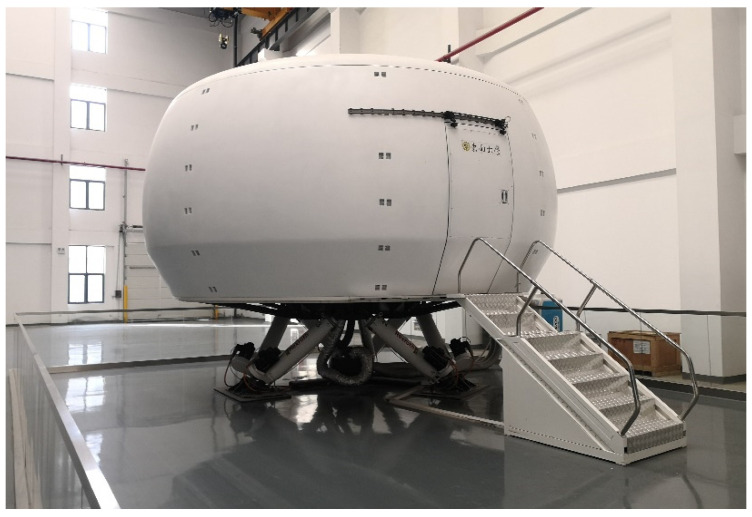
Layout of the driving simulator.

In this study, a driving simulator experiment was designed and conducted to collect overtaking data for a set of young novice drivers. The experimental equipment is a multi-degree-of-freedom motional driving simulator provided by INNOSIMULATION (see Figure 4). The virtual visibility angle is 360° and the simulated driving projection resolution is 1920 × 1080 pixels. The software programming is completed using SCANeR Studio 1.8. The experiment was conducted at the School of Transportation of Southeast University in China, which includes a test part and a formal part. In the formal one, an experiment scene was assigned to each participant randomly. Based on the collected data, we exclude those non-overtaking ones, and an in-depth analysis is conducted on the remaining overtaking data.

### 3.1. Design of the Experiment

To obtain enough overtaking data, the preliminary assumption of the simulated driving experiment scene is set to be straight roads and daytime with fine weather and high visibility. The speed limit is set to be the highest speed allowed by the road type, which is 70 km/h on outdoor intercity roads. The variables include the speed of the impeding vehicle (30 km/h or 50 km/h), the speed of the opposite vehicle (40 km/h or 60 km/h), and the type of the impeding vehicle (a passenger car or a truck). Each of these factors varies on two levels and is treated as a within-subjects variable (see Table 1). The combination of the three within-subjects factors generates eight (2^3^) treatments.

**Figure 5:**
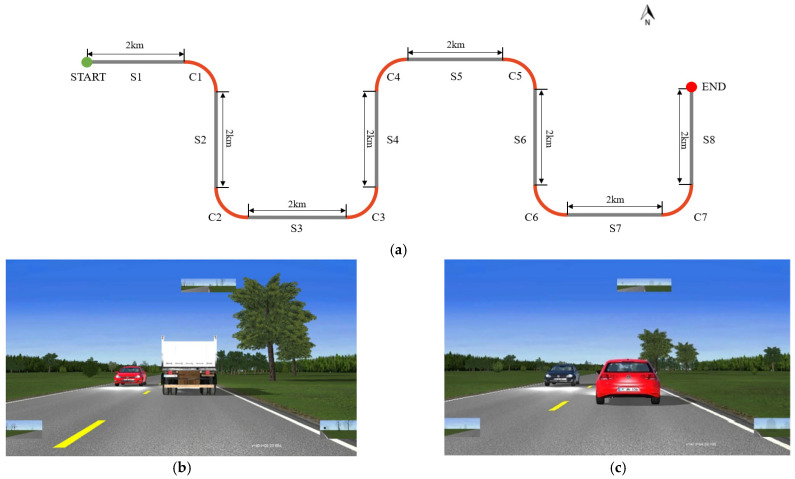
Geometric schematic of the basic scheme: (**a**) Schematic diagram of experimental road geometry; (**b**) display image (the vehicle in front is a large truck); (**c**) display image (the vehicle in front is a passenger car).

The simulated road is a two-lane intercity highway with a total length of 18 km, a lane width of 3.5 m, no shoulders, and no intersections. The experimental route consists of 8 straight line sections (S1–S8) with a length of 2 km, which are connected by 7 bends. The right-turn curve (C-1, C-4, C-5) and the left-turn curve (C-2, C-3, C-6, C-7) follow the road to coordinate the geometric shapes and to enhance the realism of the experimenter’s experience. Since it is a motional simulator, the experimenter can perceive the height difference of the curve side through vibration and weightlessness. The surrounding environment is similar to a typical rural environment, and there are no obstacles in sight. The geometric schematic of the basic scheme is shown in Figure 5.

**Figure 6:**
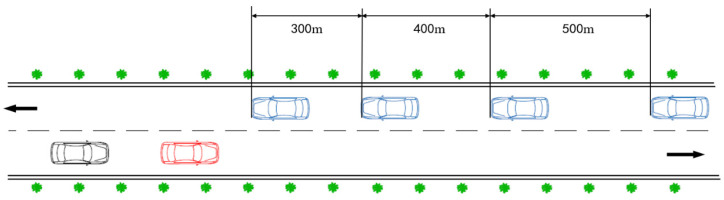
Schematic diagram of overtaking dynamic scene.

Each experimental section consists of two parts:

- Vehicle acceleration section: After entering the experimental section, a driver accelerates to his/her habitual speed under the speed limit of the highway, and the section length is set at 200 m.
- Passage section: When a vehicle crosses the white line, an impeding vehicle appears 150 m ahead with a constant speed designed in that scenario, and an opposite vehicle fleet begins to move forward with a constant speed as well. The distance between the vehicles at the opposite lane is 300, 400, and 500 m, respectively, shown in Figure 6. The driver will then choose an appropriate occasion to overtake according to his/her own needs.

### 3.2. Experimental Process

Fifty-two young novice drivers (33 males and 19 females) were recruited voluntarily from Southeast University. Two of the participants scored high in the motion sickness questionnaire (SSQ) and were therefore deleted from the sample, and three participants were extremely unskilled in driving. They did not complete the experiment and were also deleted. Therefore, the total number of participants is 47 in the end (28 males and 19 females), with an average driving experience of less than 3 years (M = 2.75, SD = 1.673). The driving time is an average of half an hour per week, which represents a lack of driving experience. The age of the subjects is between 20–30 years old (M = 23.35, SD = 2.009).

Each participant should drive the simulated scene in the test part for around 20 min before the formal experiment, so as to become familiar with the driving simulator. Participants were asked to drive in their usual driving style without any specific guidance from the technician.

Data were recorded once by the SCNeR1.8 analysis module at a frequency of 100 Hz in the formal experiment. The recorded data include the X, Y, and Z coordinates of the subject vehicle, the impeding vehicle and the opposite vehicle, the subject vehicle speed, the impeding vehicle speed, the opposite vehicle speed, vehicle’s accelerator pedal, brake pedal, turn signal, and steering amplitude data. A description of these variables is given in Table 2.

## 4. Results

**Figure 7:**
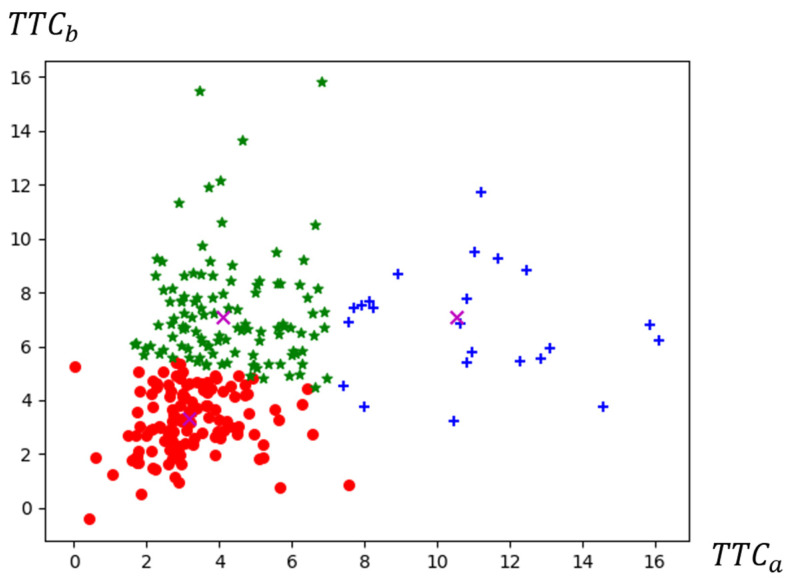
Results from the clustering analysis: red dot: category1; green star: category 2; blue cross: category 3.

By collecting the driving behavior data of these 47 participants in their 8 overtaking scenarios, we find 111 instances of not overtaking maneuvers in a total of 376 samples. We find 265 instances of overtaking maneuvers in total. Thus, the $TTC_{a}$ and $TTC_{b}$ values of a driver during his/her second phase of overtaking are computed based on Equations (6) and (8), and the FCM algorithm is then applied to group the drivers based on these two values. Three categories are identified and the results are shown in Figure 7 and Table 3. Some interesting findings are summarized below:

- The first category of the young novice drivers, representing roughly 48% of the total samples in this study, is relatively close to both the impeding and the opposite vehicle when they are turning back to their original lane. The cluster center is 3.167 for $TTC_{a}$ and 3.322 for $TTC_{b}$, indicating that this category of drivers does not reserve enough time for their second phase of overtaking, which results in relatively higher collision risk with both the impeding and the opposite vehicle when returning to the original lane. Thus, attention should be paid to this category of drivers, especially regarding their overtaking decision making at the first phase.
- The second category contains the drivers who have a similar $TTC_{a}$ value (a cluster center value of 4.111) as the first category, but a relatively larger $TTC_{b}$ value (a cluster center value of 7.082), indicating that this category of young novice drivers is quite aware of the approaching vehicle on the opposite lane, but returns to the original lane somewhat in a hurry, thus facing a relatively higher collision risk with the impeding vehicle when they are back to the lane. Such a category represents 43% of the total samples, for which safety concerns are also needed, especially on their TTC perception during the second phase of overtaking.
- Relative to the above two categories, the third category is considered as the safest one in this study. The drivers within this category keep a relatively safer distance with both the impeding and the opposite vehicle when they are driving back to the original lane. The cluster center is 10.535 for $TTC_{a}$ and 7.108 for $TTC_{b}$. However, less than 9% of the total samples are within this category, implying that not so many young novice drivers can make accurate judgments when they are at the second phase of overtaking.
- By taking all the samples into account, we can find that about half of them (47.7%) have a low $TTC_{b}$ value, while over 90% have a low $TTC_{a}$ value. Such a result implies that how to correctly avoid the collision risk with the impeding vehicle when turning back to the original lane is probably a more critical issue for young novice drivers, which should be given a higher priority for intervention.

Furthermore, to find out the potential factors that may affect the collision risk between the involved vehicles, a multi-class logistic regression analysis introduced in Section 2.4 is applied, using the three risk categories shown in Table 3 as dependent variables, and the type of the impeding vehicle (0: car; 1: truck), the speed of the opposite vehicle ($v_{OV}$), the speed of the impeding vehicle ($v_{IV}$), the average speed of the subject vehicle during the whole process of overtaking ($\bar{v_{s}}$), the duration between a driver has an intention of overtaking and he/she starts to overtake ($t_{delay}$), and the passing sight distance (PSD) as independent variables. Taking the third category, i.e., the safest category in this study, as a reference, a multi-class logistic regression model is built. We can find from the results shown in Table 4 that:

- Compared with the less risky drivers in the third category, the impeding vehicle speed $v_{IV}$ and the average overtaking speed of the subject vehicle $\bar{v_{s}}$ are statistically significant for the other two categories. Generally, a lower speed of the impeding vehicle (30 km/h vs. 50 km/h) tends to result in a lower value of $TTC_{a}$. In other words, young novice drivers are more likely to return early when they find that the speed of the impeding vehicle is low. Similarly, a higher average speed of the subject vehicle normally leads up to a riskier type of turning back (i.e., categories 1 and 2).
- Regarding $t_{delay}$, which represents the duration between a driver has an intention of overtaking and he/she starts to overtake, it has a statistically significant impact only on the first category of drivers (with both low $TTC_{a}$ and $TTC_{b}$ values). Such a result helps us to understand that because of the delay in overtaking decision making at the first phase, this type of driver has less time to complete their overtaking maneuver. Thus, a relatively higher collision risk with both the impeding and the opposite vehicle is inevitable when they return to the original lane.
- The speed of the opposite vehicle $v_{OV}$ also has a significant but negative impact on the first category of drivers. It can be explained by the fact that a higher speed of the opposite vehicle (60 km/s vs. 40 km/s) is also one of the possible reasons for this type of driver to have less time to turn back (both $TTC_{a}$ and $TTC_{b}$ values are low).
- Relative to the drivers in category 3, the impact of the type of the impeding vehicle as well as the passing sight distance on the other two categories is not significant.

## 5. Discussion

In this study, we investigated the driving behavior of young novice drivers at the second phase of overtaking on two-lane highways based on a driving simulator experiment. Some considerations regarding experiment design and data analysis are discussed below. 

First, before the design of this experiment, a questionnaire survey was conducted to better understand those potential factors that may influence drivers’ overtaking decisions and behavior. The results showed that the speed and the type of the impeding vehicle, the speed of the opposite vehicle, the distance between the opposite vehicle and the overtaking vehicle, as well as road alignment and visibility are all relevant factors. However, because the focus of this study is on the second phase of overtaking, only three of them (i.e., the speed of the impeding vehicle, the speed of the opposite vehicle, and the type of the impeding vehicle) are considered as within-subjects variables in the experiment design, while a straight road segment and daytime with fine weather and high visibility are set to stimulate as much overtaking maneuver as possible.

Second, after the simulator experiment, all the participants were also asked to fill out a questionnaire regarding their feeling about the authenticity of the simulated driving scenarios. Apart from the two drivers who suffered from motion sickness, the remaining participants all indicated that they did not feel much difference when driving in the simulator. Although positive feedback was received, we should bear in mind that the driving simulator experiments may diverge from real driving conditions and, consequently, the results may, inevitably, contain a certain bias [33]. Therefore, field data should be collected in the next step, so as to verify the results from the driving simulator.

Regarding risk assessment, the TTC values at the two critical positions were calculated and used in this study as multi-risk indications of the driver who is performing the second phase of the overtaking maneuver. Although there are two other critical positions: when returning to the lane, the front of the passing vehicle and the front of the impeding vehicle, the rear of the passing vehicle, and the rear of the opposite vehicle. The data show that they appear less frequently among novice drivers. Such a two-dimensional risk index has been proven valuable for driver classification. However, it should be noticed that overtaking is a result of the comprehensive judgment of a driver on the impeding vehicle, the opposite vehicle, and other environmental factors such as lane width. Therefore, apart from the aforementioned two critical points, there are also other types of risk, such as a collision with a highway guardrail and driving out of the lane, which are known as horizontal risk. This kind of risk, however, is not considered in this study.

## 6. Conclusions

As a complex and multi-phase maneuver, overtaking on a two-lane highway is associated with high collision risk, especially for young novice drivers. In this study, by designing and conducting a driving simulator experiment, we assess the driving risk of 47 young novice drivers during their second phase of overtaking on two-lane highways. The TTC values at the two critical positions (i.e., $TTC_{a}$ and $TTC_{b}$) are calculated from a micro-geometric point of view, and they are used as multi-risk indications of a driver’s behavior during this phase. The fuzzy C-means clustering algorithm is then applied to group all these drivers into three risk categories, and a multi-class logistic model is further developed to understand the potential factors related to the risky overtaking maneuvers at this phase. The main findings from this study are as follows:

- Only very limited young novice drivers (less than 9% of the total samples in this study) can make accurate judgments when they are at the second phase of overtaking.
- Due partly to less driving experience, young novice drivers who hesitate when making an overtaking decision at the first phase (with a larger value of $t_{delay}$), usually have a problem of reserving enough time for their second phase of overtaking, thereby frequently resulting in higher collision risk with both the impeding and the opposite vehicle when they are back to the lane (i.e., a lower value of both $TTC_{a}$ and $TTC_{b}$). Around half of the samples (47.7%) belong to this category in our study.
- When turning back to the original lane, the young novice drivers are more likely to be aware of the opposite vehicle that is approaching them, while how to correctly avoid the collision risk with the impeding vehicle at this phase is probably a more critical issue for young novice drivers, representing over 90% of all samples in this study.
- Normally, a higher average speed of the subject vehicle tends to result in a riskier type of turning back. Therefore, speed control is important for young novice drivers when they are conducting an overtaking maneuver, no matter whether at the first phase or the second.
- Regarding the impact of the impeding and the opposite vehicle, in general, young novice drivers tend to return early when they find that the speed of the impeding vehicle is low. Meanwhile, a high speed of the opposite vehicle is more likely to induce risky overtaking maneuvers.
- No significant impact of the type of the impeding vehicle and the passing sight distance on the risk categories is found.

All these findings help us gain insight into the risky behavior of young novice drivers during their second phase of overtaking on a two-lane highway, and provide valuable clues for active safety intervention such as intelligent driving assistance systems. For instance, an early warning system can be designed by setting a threshold value with the impeding vehicle when a driver is turning back to the original lane. Thus, a low TTC value with the impeding vehicle at the original lane can be avoided for those young novice drivers. Future research should then focus on how to determine such a threshold value. In addition, research on other types of risk at this phase, such as the horizontal risk mentioned in the discussion section, is worthwhile, and the collection of field data is important with the purpose of verifying the results from the driving simulator.

## Figures and Tables

**Table 1:** Variables within the experimental design group.

Impeding Vehicle Speed (IVS)							
30 km/h				50 km/h			
Impeding vehicle type				Impeding vehicle type			
Car (PC)		Truck (TR)		Car (PC)		Truck (TR)	
Opposite vehicle speed		Opposite vehicle speed		Opposite vehicle speed		Opposite vehicle speed	
40 km/h	60 km/h	40 km/h	60 km/h	40 km/h	60 km/h	40 km/h	60 km/h
30-PC-40	30-PC-60	30-TR-40	30-TR-60	50-PC-40	50-PC-60	50-TR-40	50-TR-60
1	2	3	4	5	6	7	8

**Table 2:** Description of variables.

Variable	Description
$\left(x_{SV},y_{SV},z_{SV}\right)$	Coordinates of the subject vehicle (m)
$v_{S}$	Speed of the subject vehicle (m/s)
θ	Steering angle of the subject vehicle (rad)
$\left(x_{IV},y_{IV},z_{IV}\right)$	Coordinates of the impeding vehicle (m)
$v_{IV}$	Speed of the impeding vehicle (m/s)
$\left(x_{OV},y_{OV},z_{OV}\right)$	Coordinates of the opposite vehicle (m)
$v_{OV}$	Speed of the opposite vehicle (m/s)
$\bar{v_{s}}$	The average speed of the subject vehicle when completing an overtaking (m/s)
$t_{delay}$	The duration when the driver has the idea of overtaking until he starts to overtake (s) (determined from turn signals and specific steering angle)
t	Time (s)
PSD	Passing sight distance (m)

**Table 3:** Three types of risk cluster centers.

Cluster Category	Two-Dimensional Risk Index		Number of Samples	Proportion
	$TTC_{a}$	$TTC_{b}$		
1	3.167 (M = 3.231, SD = 1.257)	3.322 (M = 3.228, SD = 1.239)	125	47.7%
2	4.111 (M = 4.269, SD = 1.408)	7.082 (M = 7.152, SD = 2.119)	113	43.1%
3	10.535 (M = 10.599, SD = 2.707)	7.108 (M = 6.472, SD = 2.456)	23	8.8%

**Table 4:** Results from the multi-class logistic model.

	Factors	B	Standard Error	Wald	Significance	Exp (B)
1	$\bar{v_{s}}$	0.675	0.178	14.287	**0.000**	1.963
	$t_{delay}$	0.133	0.064	4.316	**0.038**	1.143
	[type = 0]	0.760	0.591	1.649	0.199	2.137
	$[v_{IV}$= 30]	3.364	1.279	6.915	**0.009**	28.918
	$[v_{OV}$= 40]	−1.232	0.613	4.036	**0.045**	0.292
	[PSD = 1]	2.123	2.170	0.957	0.328	8.354
	[PSD = 2]	2.096	1.885	1.235	0.266	8.130
	[PSD = 3]	0.490	1.866	0.069	0.793	1.632
2	$\bar{v_{s}}$	0.560	0.167	11.300	**0.001**	1.751
	$t_{delay}$	0.072	0.059	1.513	0.219	1.075
	[type = 0]	0.492	0.561	0.770	0.380	1.635
	$[v_{IV}$= 30]	3.846	1.276	9.090	**0.003**	46.789
	$[v_{OV}$= 40]	−0.173	0.574	0.091	0.763	0.841
	[PSD = 1]	−3.246	1.971	2.711	0.100	0.039
	[PSD = 2]	−0.806	1.608	0.251	0.616	0.447
	[PSD = 3]	−1.254	1.563	0.644	0.422	0.285

The reference category is: 3 (the safest category in this study). Bold items are the statistically significant values (*p* < 0.05).

## Data Availability

The data presented in this study can be provided by the authors upon requests.
